# Signaling proteins in HSC fate determination are unequally segregated during asymmetric cell division

**DOI:** 10.1083/jcb.202310137

**Published:** 2024-06-14

**Authors:** Amol Ugale, Dhanlakshmi Shunmugam, Lokesh G. Pimpale, Elisabeth Rebhan, Manuela Baccarini

**Affiliations:** 1 Department of Microbiology, Max Perutz Labs Vienna, University of Vienna, Immunobiology and Genetics, Vienna, Austria; 2 Vienna BioCenter PhD Program, Doctoral School of the University of Vienna and Medical University of Vienna, Vienna, Austria; 3 HeartBeat.bio AG, Vienna, Austria

## Abstract

Hematopoietic stem cells (HSCs) continuously replenish mature blood cells with limited lifespans. To maintain the HSC compartment while ensuring output of differentiated cells, HSCs undergo asymmetric cell division (ACD), generating two daughter cells with different fates: one will proliferate and give rise to the differentiated cells’ progeny, and one will return to quiescence to maintain the HSC compartment. A balance between MEK/ERK and mTORC1 pathways is needed to ensure HSC homeostasis. Here, we show that activation of these pathways is spatially segregated in premitotic HSCs and unequally inherited during ACD. A combination of genetic and chemical perturbations shows that an ERK-dependent mechanism determines the balance between pathways affecting polarity, proliferation, and metabolism, and thus determines the frequency of asymmetrically dividing HSCs. Our data identify druggable targets that modulate HSC fate determination at the level of asymmetric division.

## Teaser

Ugale et al. demonstrate that CDC42, ERK, and mTORC1 signaling are polarized in premitotic hematopoietic stem cells and unequally segregated during asymmetric cell division. A CDC42/ERK/mTORC1 pathway maintains HSC polarity and balances symmetric and asymmetric cell division.

## Graphical Abstract

**Figure:**
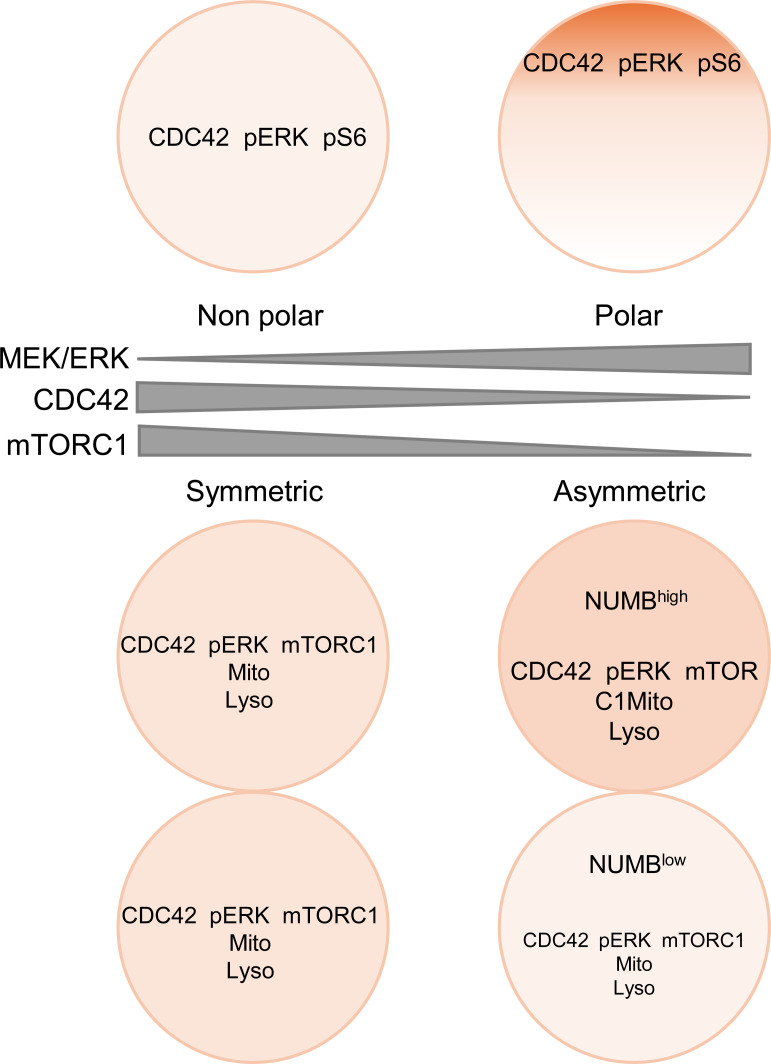

## Introduction

The production of blood cells throughout life depends on the fitness of the hematopoietic stem cell (HSC) compartment. HSCs are mostly quiescent (Bernitz et al., 2016; Takizawa et al., 2011; Wilson et al., 2008) but when activated gradually transition from a dormant state (Cabezas-Wallscheid et al., 2017; Wilson et al., 2008) to a fully activated state in which they cycle and differentiate. HSCs exist in fluent transcriptional states. The proportion of HSCs in each state can be altered by external stimuli such as emergency hematopoiesis (Wilson et al., 2008). HSCs also show great transcriptional heterogeneity, an intrinsic property that may dictate the response of HSC subsets to specific signals inducing the activation state (Fast et al., 2021). The identity of the signals involved in the transition is starting to be unraveled. The mTOR pathway promotes HSC differentiation at the expense of self-renewal (Chen et al., 2008; Kharas et al., 2010b; Rodgers et al., 2014; Yilmaz et al., 2006), and mechanistic target of rapamycin (mTOR) inhibitors such as rapamycin are considered rejuvenating agents for many stem cell types (Neves et al., 2017). The extracellular-signal-regulated kinase (ERK) pathway controls the balance between differentiation and return to quiescence during emergency hematopoiesis, primarily by regulating mTORC1 activation (Baumgartner et al., 2018).

The rate-limiting step after which the HSC progeny is rapidly amplified to meet the demands of hematopoiesis is the transition from HSC self-renewal to differentiation in multipotent progenitors with intermediate or short-term reconstitution potential. Signaling events such as the ones mentioned above might play a role in the transition; however, this abrupt change of fate is also implemented through asymmetric cell division (ACD), the process by which fate of the daughter cells is determined by the unequal inheritance of cell components. ACD has been recently shown to occur in both mouse (Loeffler et al., 2019) and human HSC (Loeffler et al., 2021), and an important role for both lysosomes and mitochondria in the determination of daughter cell fates has been recognized. Organelle inheritance, intracellular signaling pathways, and metabolism are linked at many levels, with lysosomes working as a platform for mTORC1 signaling (Ballabio and Bonifacino, 2020) and mTORC1, in turn, inducing mitochondrial biogenesis and reducing autophagy (Chandel et al., 2016; Ito and Suda, 2014). In addition, the small GTPase CDC42 is a critical regulator of HSC mode of division in aging, with an increase in CDC42 signaling linked to more symmetric divisions and reduced repopulation capacity (Florian et al., 2018). CDC42 regulates polarity and, by extension, the mode of division of HSC via its interactors BORG4 and SEPTIN 7, both necessary for HSC function during stress hematopoiesis (Kandi et al., 2021). Finally, epigenetic mechanisms play an important role in the functional outcome of stem cell division (Florian et al., 2012, 2018), where different classes of epigenetic modifiers contribute to HSC self-renewal as well as lineage specification and differentiation into various cell types.

Understanding the mechanism(s) balancing HSC quiescence, self-renewal, and differentiation is crucial to optimize HSC expansion for therapeutic strategies and rejuvenation of aging HSCs. Additionally, to be able to manipulate the system, we must learn more about the rate-limiting steps determining the fitness of the HSC compartment and about the signaling pathways that regulate them. Here, we explore the possibility that spatial segregation of crucial signaling pathways during ACD is one of these steps and that the modulation of this step affects HSC fate at this early level.

## Results

### Segregation of cell fate determinants, organelles, and signaling molecules during the first cell division of HSC in culture

**Figure S1.**
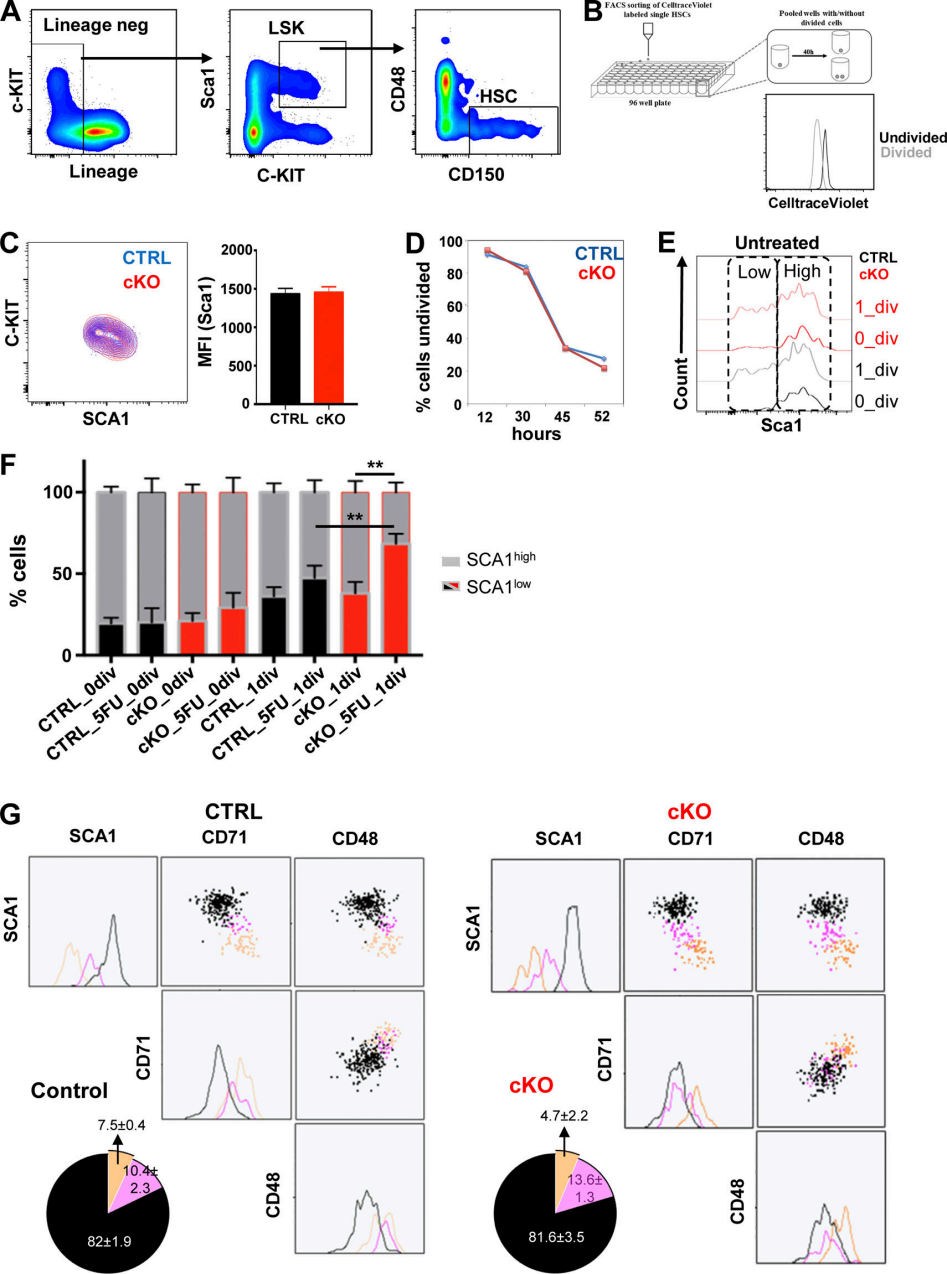
**Characteristics of HSCs analyzed in this study.** Related to Fig. 1. **(A)** Schematics of FACS gating strategy for HSCs sorting (LSK CD48^−^CD150^+^). **(B)** Schematic FACS analysis strategy for validation of CellTrace Violet labeling dilution in HSCs. The plot shows the CellTrace Violet content of undivided (black) and divided cells (gray). **(C)** SCA1 and C-Kit expression in CTRL versus cKO HSCs. Left, representative plot; right, bar graph showing the mean ± SD fluorescence intensity (MFI) of SCA1 (*n* = 3). **(D)** Percentage of control and MEK1-cKO HSCs that divided over time, assessed by tracking single HSCs (CTRL: *n* = 173 and cKO: *n* = 152). **(E)** Representative FACS analysis of SCA1 expression in CTRL and cKO HSCs after 42 h in culture. HSCs were isolated from untreated mice. Cells that have not divided during this time are depicted in black (CTRL) or red (cKO). Cells that have undergone one cell division are depicted in gray (CTRL) or light red (cKO). **(F)** Stacked bar charts depicting the percentage of SCA1^high^ and SCA1^low^ from CTRL (black) versus MEK1-cKO (red) HSC cultures, isolated from untreated or 5-FU–treated mice. **(G)** Representative clustering analysis of SCA1, CD71, and CD48, and expression in undivided cells from CTRL (left panel) and cKO (right panel) HSC cultures. HSCs were isolated from mice recovering from one 5-FU injection. Three clusters were generated using FlowSOM: cluster 1 (black), cluster 2 (pink), cluster 3 (yellow). The frequency of cells in the corresponding cluster is depicted in the pie charts that combine three independent experiments. The mean (± SD) percentage of cells in each cluster is indicated.

**Figure 1.**
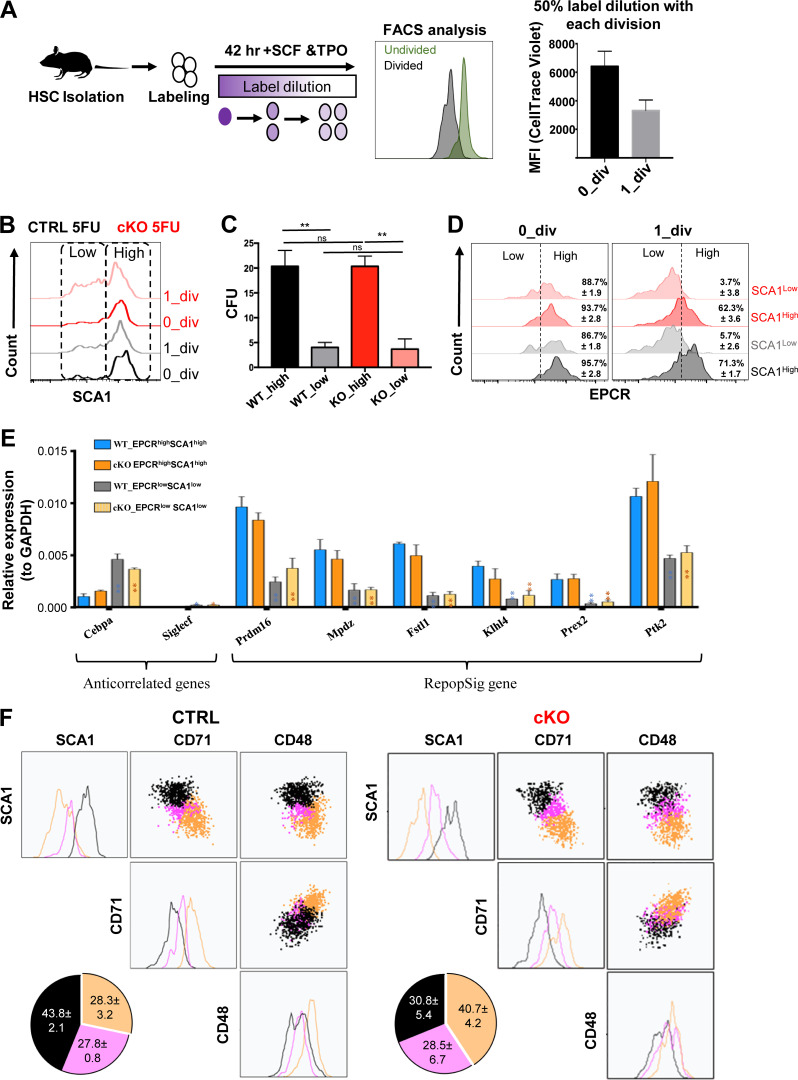
**MEK1 ablation increases the number of SCA1**^**low**^ **cells generated in HSC cultures. (A)** Schematic representation of the experimental setup for FACS analysis of HSCs cultures. The bar graph depicts the mean fluorescence intensity (MFI) of CellTrace Violet 16 h after labeling (labeling reference, 0_div) and the label dilution upon cell division (1_div). **(B)** Representative FACS analysis of SCA1 expression in CTRL and cKO HSCs after 42 h in culture. Cells that have not divided during this time are depicted in black (CTRL) or red (cKO). Cells that have undergone one cell division are depicted in gray (CTRL) or light red (cKO). **(C)** CFUs derived from 1_div SCA1^high^ and SCA1^low^ cells generated in CTRL and cKO HSC in LTC-IC assay (*n* = 3). **(D)** Representative FACS analysis of EPCR expression in SCA1^high^ and SCA1^low^ from CTRL and cKO HSC cultures after 42 h in culture. Cells that have not divided during this time are depicted in black (CTRL) or red (cKO). Cells that have undergone one cell division are depicted in gray (CTRL) or light red (cKO). **(E)** qPCR analysis of RepopSig genes and anti-correlated genes expressed in SCA1^high^ EPCR^high^ and SCA1^low^ EPCR^low^ cells generated in CTRL and cKO cultures after one division. **(F)** Representative clustering analysis of SCA1, CD71, CD48, and expression in 1_div cells from CTRL (left panel) and cKO (right panel) HSC cultures. HSCs were isolated from mice recovering from one 5-FU injection. Three clusters were generated using FlowSOM: cluster 1 (black), cluster 2 (pink), cluster 3 (yellow). The frequency of cells in the corresponding cluster is depicted in the pie charts as mean ± SD of three independent experiments. **(A, D, and E)** Error bars represent the SD of the mean. **P < 0.01, according to unpaired *t* test comparing CTRL to cKO. **(F)** Black and yellow clusters: P < 0.0001 according to Fisher’s exact test comparing CTRL and cKO clusters.

To investigate how cultured HSCs behave during initial divisions, we employed FACS-based cell division tracking of cells isolated using a well-established FACS gating strategy (Fig. S1 A; Baumgartner et al., 2018) using CellTrace Violet labeling combined with markers reporting on HSC differentiation and metabolic state (Fig. 1 A). Cells showing a 50% dilution of the initial CellTrace Violet reference were assumed to have divided once (Fig. 1 A). The reliability of the label dilution was confirmed by single-cell-sorting HSCs in 96-well U-bottom plates and culturing them for 40 h, after which time wells with two cells (divided cells) and wells with one undivided cell were pooled and their respective CellTrace Violet content analyzed by FACS. The profiles (Fig. S1 B) recapitulate the label dilution shown in Fig. 1 A.

To correlate the behavior of HSCs with known impacts of perturbation on cell signaling and cell fate, we have compared control HSC with mitogen-activated protein kinase (MEK1)–deficient HSC (cKO). MEK1 ablation promotes signaling through both the ERK and the PIP3/mTORC1 pathway in the population of activated HSCs and skews the balance between activated HSCs that return to quiescence and those that underwent further differentiation toward this latter population. In vivo, this leads to reduced HSC numbers and loss of label-retaining cells in aging mice, and to HSC exhaustion under stress conditions such as transplantation or chronic myelotoxicity (Baumgartner et al., 2018). Control (CTRL) and cKO HSCs showed similar expression levels of the stemness-associate marker SCA1 and in C-Kit (Fig. S1 C). Microscopic examination of single HSCs of either genotype in 96-well plates confirmed that first divisions induced by cytokine stimulation (stem cell factor [SCF], 10 ng/ml; thrombopoietin [TPO], 10 ng/ml) happened mostly in a window between 30 and 42 h (Fig. S1 D) (Florian et al., 2018).

Most HSCs are in G_0_, and it has been questioned whether deeply dormant HSCs significantly contribute to steady-state hematopoiesis. The low frequency of cycling cells makes it difficult to study the events accompanying the first cell division. We have therefore used 5-Fluorouracil (5-FU) treatment to induce HSC exit from G_0_ and their transition from the dormant into the activated state (Wilson et al., 2008). In addition, 5-FU treatment also gives us a handle on the signaling mechanisms that impact the early steps of differentiation because it unmasks the phenotype of the MEK1 KO cells (Baumgartner et al., 2018). Consistent with this, 5-FU treatment in vivo led to the production of more SCA1^low^ HSCs upon initial cell division in cultures of either genotypes, and the effect was significantly greater in MEK1-cKO than in control cultures (Fig. 1 B and Fig. S1, E and F). HSCs that had not divided in this period of time expressed SCA1 at the same level (Fig. 1 B and Fig. S1, E and F). In functional terms, SCA1^high^ cells had higher colony-forming capacity compared with the SCA1^low^ cells in long-term culture–initiating cell (LTC-IC) assay, irrespective of the genotype (Fig. 1 C). Both CTRL and cKO SCA1^high^ cells also expressed high levels of endothelial protein C receptor (EPCR) (Che et al., 2022; Zhang et al., 2024), although cKO cultures generated a smaller proportion of SCA1^high^, EPCR^high^ cells compared with CTRL cultures (Fig. 1 D). SCA1^high^, EPCR^high^ cells of both phenotypes were characterized by the expression of the repopulation signature genes (RepopSig) that identify functional HSCs from multiple cellular states (Che et al., 2022) (Fig. 1 E). Together, these data confirm that we can use SCA1 as an indicator of residual HSC activity in further experiments and show that MEK1 ablation affected the frequency of SCA1^high^ EPCR^high^ cells persisting after the first HSC division in culture, but does not impact the colony forming ability or the expression of the RepopSig genes (Fig. 1, C–E).

We next used surface markers associated with differentiation (CD71 and CD48) (Loeffler et al., 2019), to characterize the output of control and MEK1-cKO. Two-dimensional representation of the landscape of HSCs retaining the CellTrace Violet label (undivided cells) using FlowSOM meta-cluster analysis (Van Gassen et al., 2015) revealed clusters that indicated a gradual transition from the most primitive to the more activated/differentiated population (Fig. S1 G). Cluster 1 comprised SCA1^high^CD71^low^CD48^low^ cells. Intermediate levels of SCA1, CD71, and CD48 expression were observed in cluster 2. In contrast, cluster 3 comprised predominantly SCA1^low^CD71^high^ and CD48^high^ cells. Cluster 1 dominated the undivided population of either genotype, confirming that we seeded HSC populations with comparable profiles.

**Figure S2.**
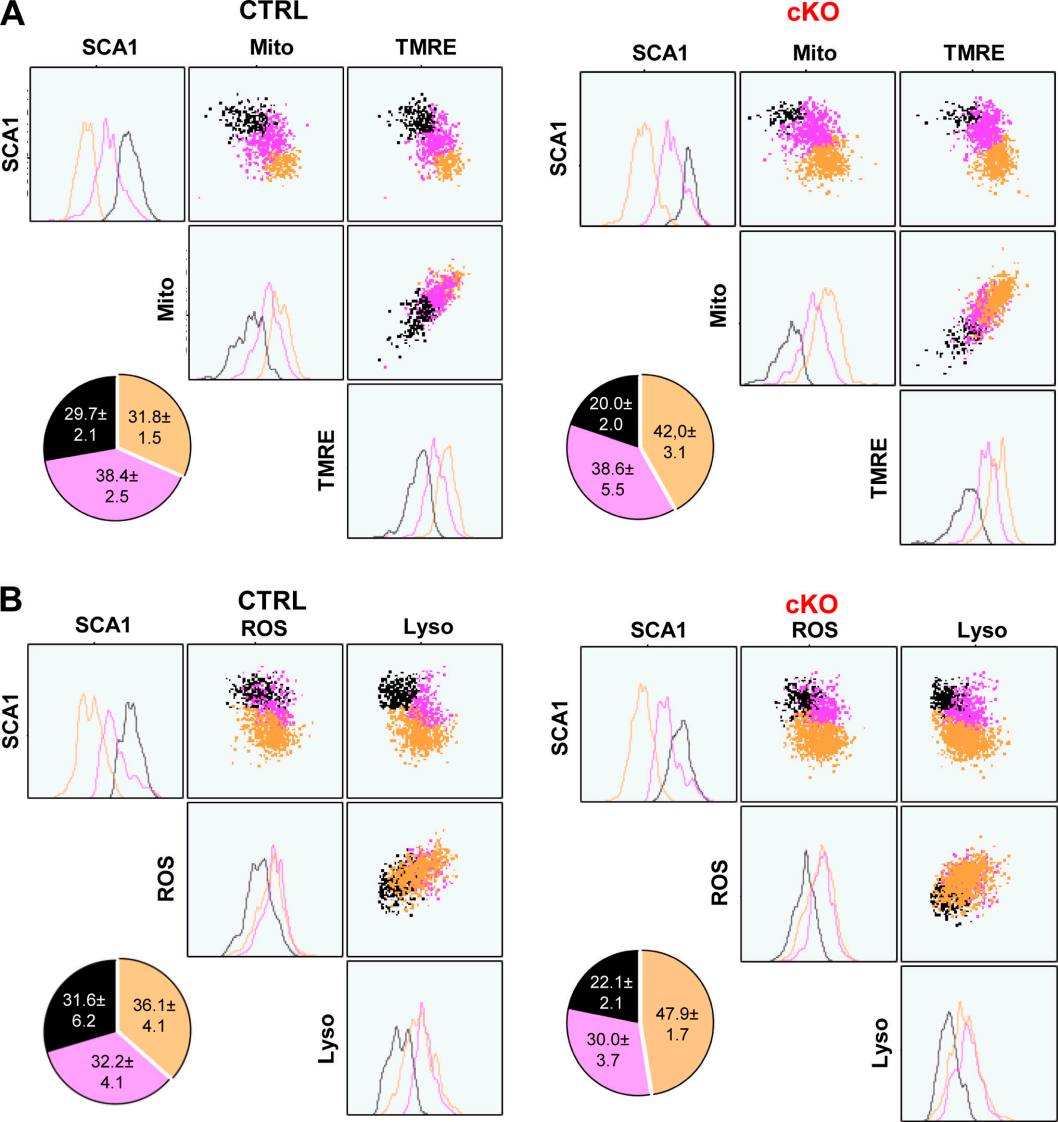
**MEK1 ablation increases the frequency of metabolically active HSCs generated in culture.** Related to Fig. 1. **(A and B)** Representative clustering analysis of Sca1, MitoTracker, tetramethylrhodamine ethyl ester (TMRE) (A) and for Sca1, ROS, LysoTracker (B) in 1_div cells from CTRL (left panel) and cKO (right panel) HSC cultures. HSCs were isolated from mice recovering from one 5-FU injection. The pie charts depict the frequency of cells in the corresponding cluster in three independent experiments. The mean (± SD) percentage of cells in each cluster is indicated. For A and B, P < 0.0001 according to Fisher’s exact test comparing CTRL and cKO black and yellow clusters.

The same three clusters could be observed in HSCs allowed to divide once in culture (i.e., containing about half of the original CellTrace Violet label). After division, however, the percentage of cells in cluster 1 was reduced (about half in the CTRL cultures), while the percentage of cells in cluster 3 increased. This cluster distribution is consistent with the well-established concept that hematopoiesis is a continuum process (Laurenti and Göttgens, 2018; Velten et al., 2017). Importantly, although the marker distribution remained similar in control and cKO HSC cultures, the latter generated a significantly higher proportion of cluster 3 cells (Fig. 1 F). This agrees with our previous observation that MEK1-cKO HSCs have bland phenotypes exacerbated by stress (Baumgartner et al., 2018). A picture similar to the one in Fig. 1 F emerged from the analysis of organelle/metabolic markers associated with HSC differentiation (Loeffler et al., 2019). Mitochondrial content and membrane potential (measured using MitoTracker dye and tetramethylrhodamine methyl ester retention, which correlates with oxidative phosphorylation; Perry et al., 2011), as well as the production of reactive oxygen species (ROS) and lysosomal content, measured using the pH-sensitive fluorescent probe LysoTracker, correlated negatively with SCA1, increasing from cluster 1 to cluster 3 irrespectively of the genotype of the HSC cultures. However, MEK1-cKO cultures generated a higher number of cluster 3 cells (Fig. S2, A and B).

Thus, SCA1 expression correlated inversely with markers of differentiation and metabolic activity. In all cases, the MEK1-cKO cultures generated more cells with low SCA1 expression and higher differentiation markers/metabolic activity compared with control cultures (Fig. 1, C–E; and Fig. S2, A and B).

**Figure S3.**
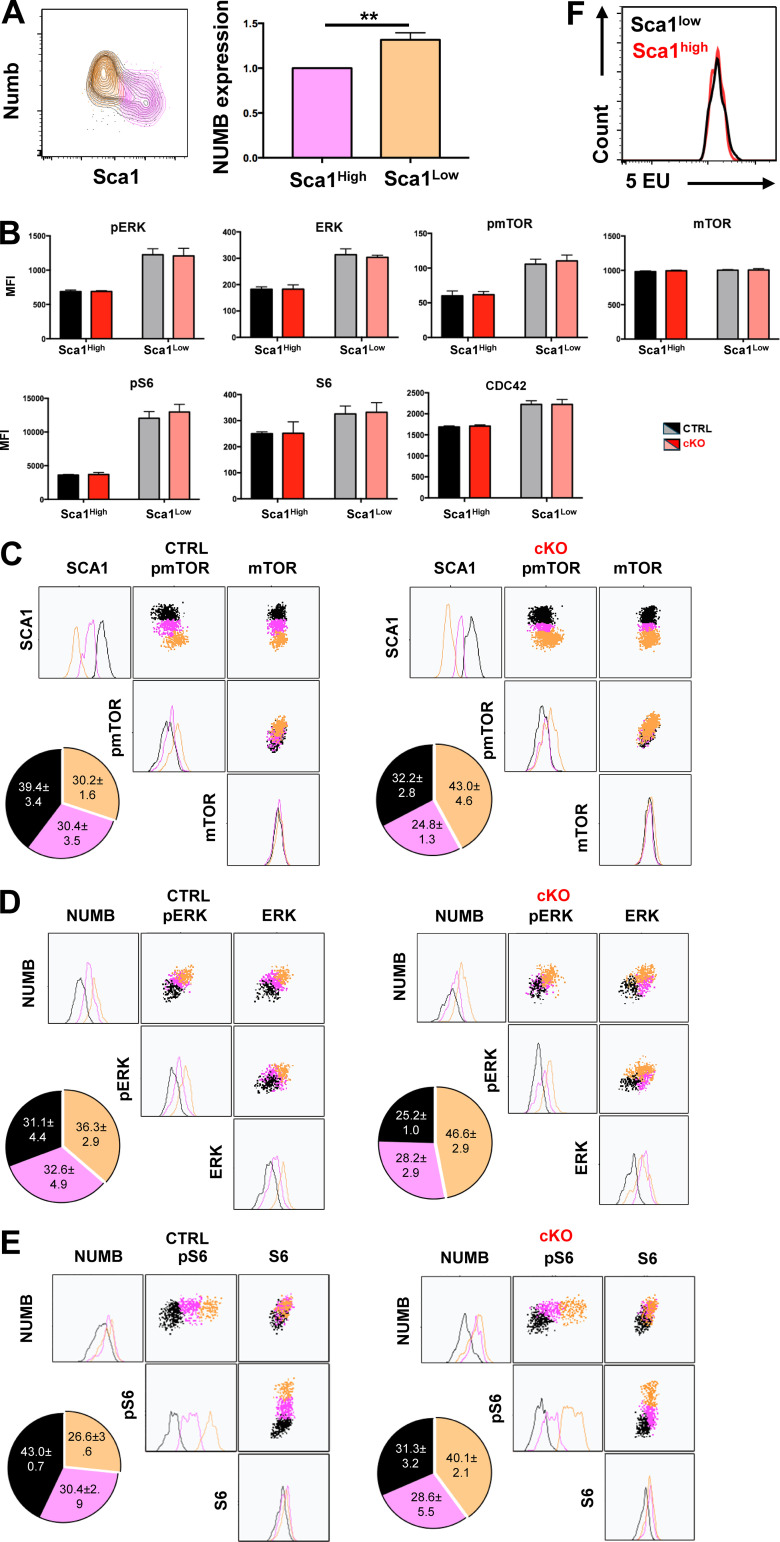
**MEK1 ablation increases the frequency of HSCs with higher signaling activity.** Related to Fig. 2. **(A)** Representative FACS plot (left) and bar graph (right) showing Numb expression in Sca1^high^ and Sca1^low^ cells. **(B)** Bar graphs showing the relative mean fluorescence intensity (MFI) of signaling proteins in SCA1^high^ and SCA1^low^ cells generated in CTRL versus cKO HSC cultures (*n* = 3). HSCs were isolated from mice recovering from one 5-FU injection. **(C–E)** Representative clustering analysis of 1_div cells from CTRL (left panel) or cKO (right panel) HSC cultures. SCA1 expression was combined with pmTOR and total mTOR (C); NUMB expression was combined with pERK and total ERK (D) or pS6 and total S6 (E). **(F)** FACS plot showing the levels for 5-EU (transcription rate) in Sca1^high^ and Sca1^low^ cultured HSCs. HSCs were isolated from mice recovering from one 5-FU injection. The pie charts depict the frequency of cells in the corresponding cluster in three independent experiments. The mean (± SD) percentage of cells in each cluster is indicated. For C–E, P < 0.0001 according to Fisher’s exact test comparing CTRL and cKO black and yellow clusters.

These experiments demonstrate that we can use this system to correlate the distribution of differentiation markers with that of organelles in the initial division of HSC in culture and that the system faithfully reports the impact of signaling perturbations on the output of the division. We therefore went on to investigate whether the levels of activity of key signaling pathways could be correlated with the expression of cell fate markers in HSCs dividing in culture. The cell fate markers used were SCA1, high in less differentiated and low in more differentiated cells, and NUMB, whose expression is inversely correlated to SCA1 expression and therefore indicates more differentiated cells (Fig. S3, A and B). Signaling activity was analyzed by phosflow analysis, and CellTrace Violet dilution was used to focus on initial division.

**Figure 2.**
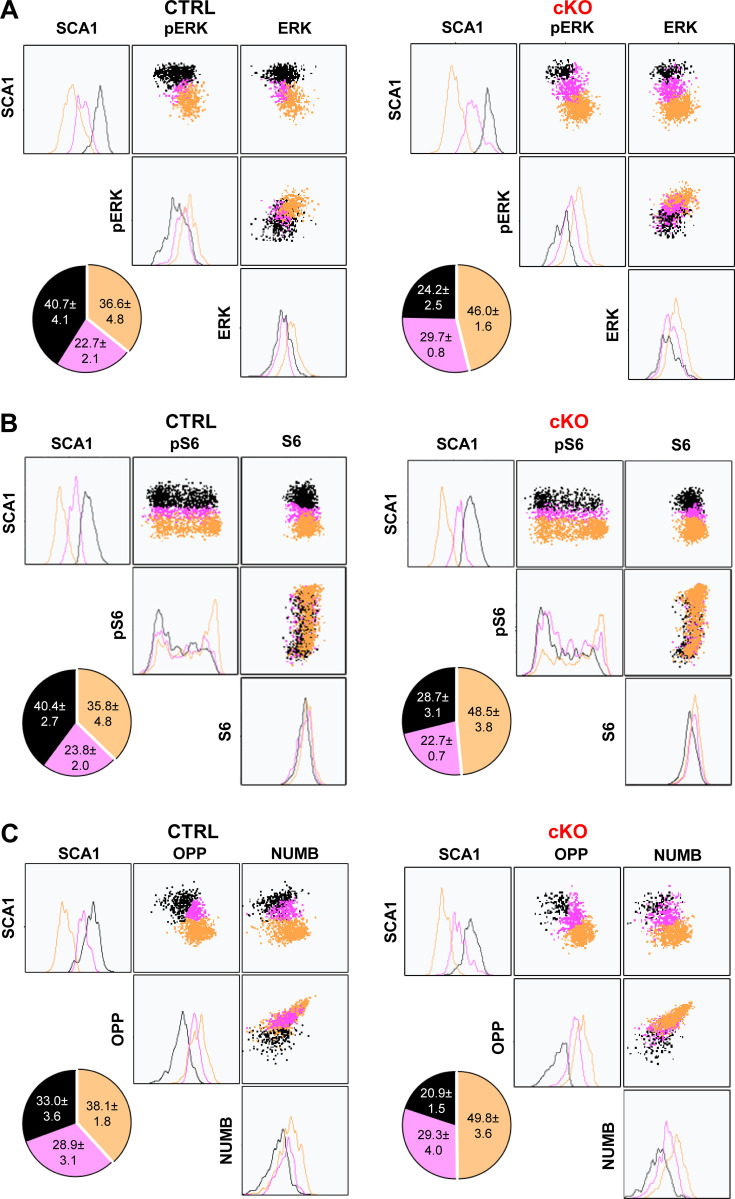
**MEK1 ablation increases the frequency of HSCs with higher signaling activity. (A–C)** Representative clustering analysis of divided cells from CTRL (left panel) or cKO (right panel) HSC cultures. SCA1 expression was combined with pERK and total ERK (A) S6 and pS6 (B) or OPP (as a measure of active translation) and NUMB (C). HSCs were isolated from mice recovering from one 5-FU injection. The pie charts depict the frequency of cells in the corresponding cluster in three independent experiments. The mean (± SD) percentage of cells in each cluster is indicated. P < 0.0001 according to Fisher’s comparing CTRL and cKO black and yellow clusters.

MEK1 ablation did not impact the abundance and phosphorylation of signaling proteins, which was strongly increased in more differentiated, SCA1^low^ HSCs of either genotype (Fig. S3 B). Unbiased clustering again revealed a continuum of transitioning cells from cluster 1 (high levels of SCA1, low pERK) to clusters 2 and 3, which mostly consisted of cells progressively losing SCA1 expression with increasing pERK content, indicating higher signaling activity in less undifferentiated cells. MEK1-cKO cultures generated more cells in the SCA1^low^, pERK^high^ cluster 3 (Fig. 2 A).

Next, we assessed the activation of mTORC1, which regulates protein translation, cellular lysosomal content, and ROS metabolism, and is impacted by MEK1 ablation. Activation was assessed by monitoring phosphorylation of mTOR on the S6K1-dependent site S2448 (Chiang and Abraham, 2005; Holz and Blenis, 2005) and of the mTORC1 downstream target S6 on S235/236. Like ERK phosphorylation, pmTOR S2448 (Fig. S3 C) and pS6 (Fig. 2 B) were highest in SCA1^low^ cells and lower in the SCA1^high^ cluster, indicating that signaling, just like metabolic activity, is weaker in less differentiated cells and stronger in cells primed for differentiation. Consistent with the inverse correlation between SCA1 and NUMB, NUMB expression correlated positively with greater signaling strength (Fig. S3, D and E).

S6 phosphorylation correlates with translational activity, which is low in HSCs (Loeffler et al., 2019). To measure the translational activity of the HSC populations generated in vitro, we used click-it chemistry to detect O-propargyl-puromycin (OPP) incorporation into nascent proteins. OPP incorporation paralleled S6 phosphorylation and was highest in SCA1^low^, NUMB^high^ cells. In contrast, RNA transcription, detected by 5-ethynyl uridine incorporation into nascent transcripts, was comparable in SCA1l^ow^ and SCA1^high^ cells (Fig. S3 F). Thus, increased protein translation in SCA1^low^, NUMB^high^ cells correlated perfectly with increased mTORC1 signaling in this population. Similar to what we showed for ERK phosphorylation, clustering of the data revealed an inverse correlation between SCA1 expression, highest in cluster 1, and pS6, OPP, and NUMB, progressively increasing in cluster 3 (Fig. 2, B and C). Compared with controls, MEK1-cKO HSC cultures consistently yielded a significantly higher fraction of cluster 3 cells, SCA1^low^ cells with higher pS6, and translation activity (Fig. 2, B and C).

### Signaling activity unequally segregates during asymmetric division

**Figure S4.**
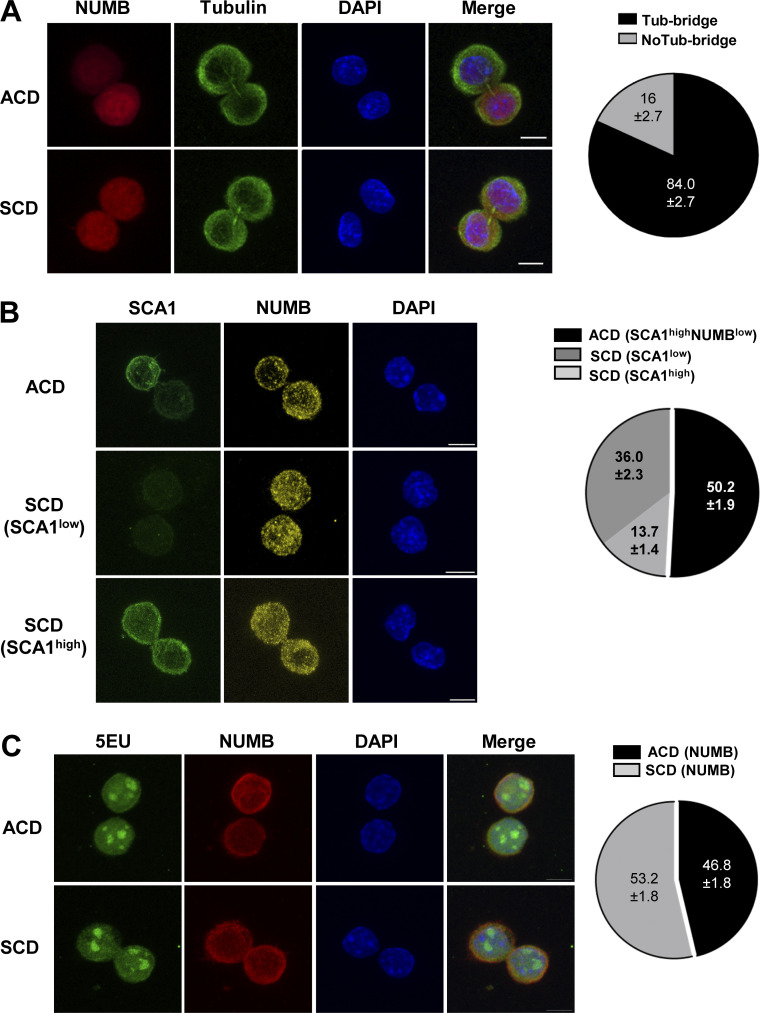
**Frequency of paired daughter cells and transcriptional activity in HSC doublets.** Related to Fig. 3. **(A)** Representative immunofluorescence images of the distribution of NUMB combined with an anti-tubulin antibody to visualize the tubulin bridges connecting daughter cells. The percentage of cells with tubulin bridge is shown in the pie chart, with a total of 219 cells. Pie chart depicting the percentage of asymmetric and symmetric divisions based on NUMB in paired daughter cells from CTRL HSCs cultures. **(B)** Representative immunofluorescence images showing the distribution of SCA1 and NUMB in paired daughter HSCs. Pie chart depicting the percentage of asymmetric and symmetric divisions in paired daughter cells from CTRL HSCs cultures. SCA1 and NUMB anticorrelated in 100% of the ACDs. NUMB was symmetrically segregated in both SCA1^high^ and SCA1^low^ daughter cells (mean percentage ± SD; 80 cells total). **(C)** Representative immunofluorescence images of the distribution of 5-EU and NUMB in CTRL daughter HSC. Pie charts depicting the percentage of asymmetric and symmetric divisions based on NUMB in paired daughter cells from CTRL HSCs cultures. Segregation of 5-EU was not observed (100% SCD) (130 cells total). Images were acquired on a Zeiss LSM 980 inverse confocal microscope using a Plan-Apochromat 63× oil objective (NA 1.4) (bar = 5 µm). The chart shows the results of three independent experiments (mean percentage ± SD).

**Figure 3.**
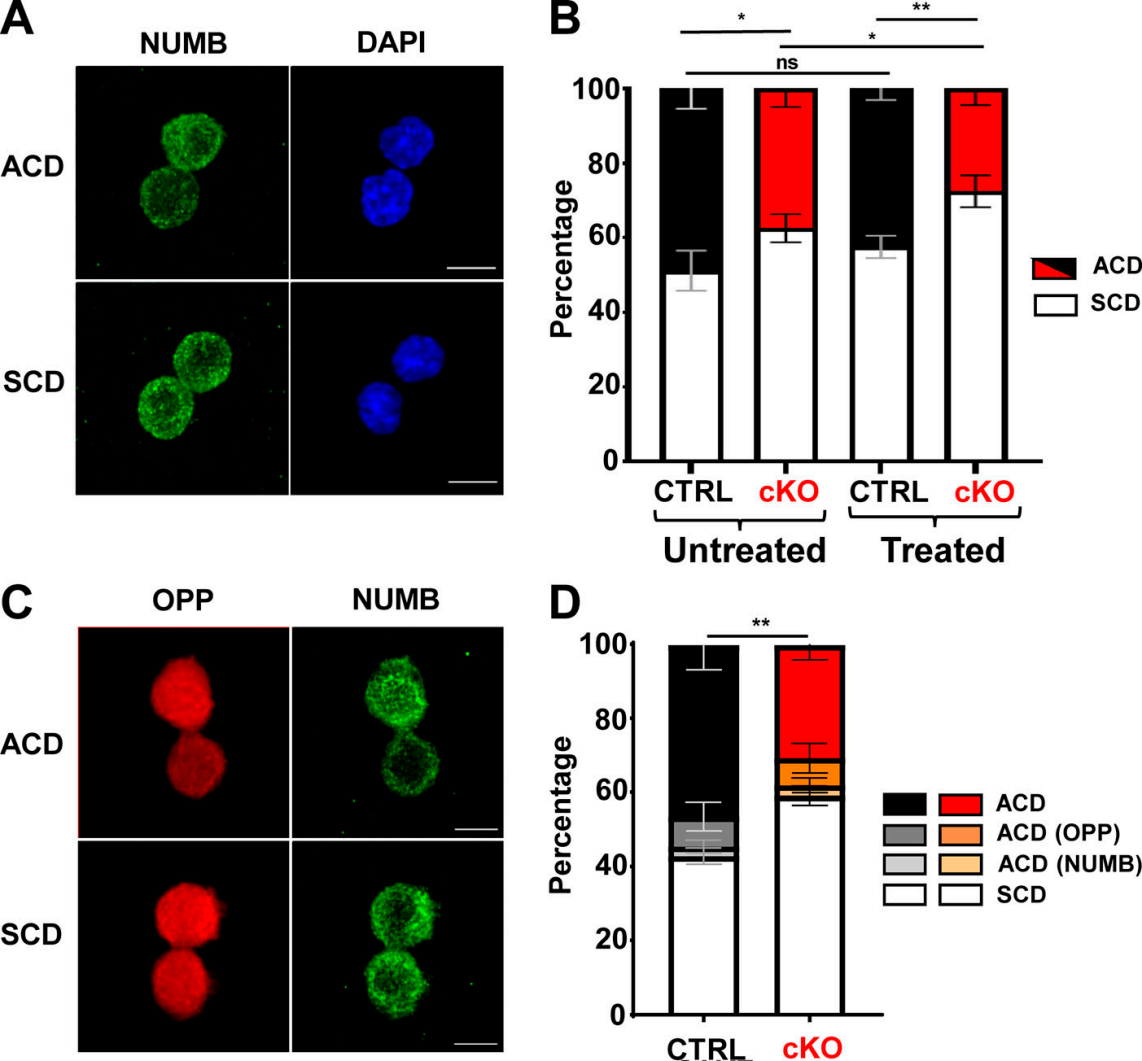
**MEK1 ablation reduces the frequency of asymmetric cell divisions in cultured HSCs. (A)** Representative immunofluorescence images showing the distribution of NUMB in paired daughter HSCs. Division is considered asymmetric when the ratio of NUMB in the paired daughter cells is > 1.5 (bar = 5 µm). **(B)** Stacked bar charts depicting the percentage of asymmetric and symmetric divisions in paired daughter cells from CTRL (black) versus MEK1-cKO (red) HSCs cultures. HSCs were isolated from untreated (left) or 5-FU–treated animals (right). **(C)** Representative immunofluorescence images of the distribution of OPP and NUMB in paired daughter HSCs (bar = 5 µm). **(D)** Stacked bar charts depicting the percentage of cells with asymmetric distribution of NUMB, OPP, or both in paired daughter CTRL (black) or MEK1 cKO (red) HSCs isolated from 5-FU–treated animals. The charts combine the results of three independent experiments (mean percentage ± SD; ≈200–250 cells total). Images were acquired on a Zeiss LSM 980 inverse confocal microscope using a Plan-Apochromat 63× oil objective (NA 1.4). *P < 0.05 and **P < 0.01.

To investigate whether the unequal cluster distribution in control and cKO HSC cultures correlated with the asymmetric inheritance of cell fate determinants at the single-cell level, we performed paired daughter immunofluorescence assays on sparsely seeded HSCs. The high number of fluorophores necessary for our experiments precluded the inclusion of tubulin bridge staining in every assay. Therefore, we first established that our experimental setup reliably scored paired HSC daughters engaged in either symmetric cell division (SCD) or ACD (84% ± 2.7; Fig. S4, A and B). In these experiments, we used NUMB, which has been shown to be asymmetrically inherited during HSC division (Wu et al., 2007; Zimdahl et al., 2014), as a cell fate marker. NUMB was chosen over the surface marker SCA1, used as a proxy for stemness in FACS, because as an intracellular protein, it can be more reliably used in routine immunofluorescence analysis under the experimental conditions necessary to stain intracellular signaling proteins. In symmetric divisions, we observed a minority of SCA1 ^high^ daughter cells, which possibly resulted from self-renewal, and a majority of SCA1^low^ daughter cells, which might have been generated by direct differentiation (Fig. S4, C and D), with the caveat of the antigen’s stability under the staining conditions. We were able, however, to show that SCA1 and NUMB segregated into opposite daughter cells during ACD (Fig. S4, C and D), as predicted by FACS analysis (Fig. S3 A), and therefore proceeded with the analysis using NUMB as a cell fate marker. Under these conditions, we could show that asymmetric segregation of NUMB was less frequent in HSCs isolated from untreated cKO mice compared with CTRL mice (Fig. 3, A and B). In line with our previous observations (Baumgartner et al., 2018) and with the FACS analysis (Fig. 1 B and Fig. S1 F), this phenotype was exacerbated when HSCs were isolated from mice recovering from myelotoxicity (Fig. 3, A and B). The same pattern was observed using NUMB as a cell fate marker or OPP as an indicator of translational activity. These experiments also provided direct evidence of stronger translation activity in NUMB^high^ daughter cells during asymmetric division, as predicted by the FACS data (Fig. 2 C). While OPP and NUMB co-segregated in most of the divisions observed, a minor fraction of cells displayed either OPP or NUMB asymmetry (Fig. 3, C and D).

**Figure 4.**
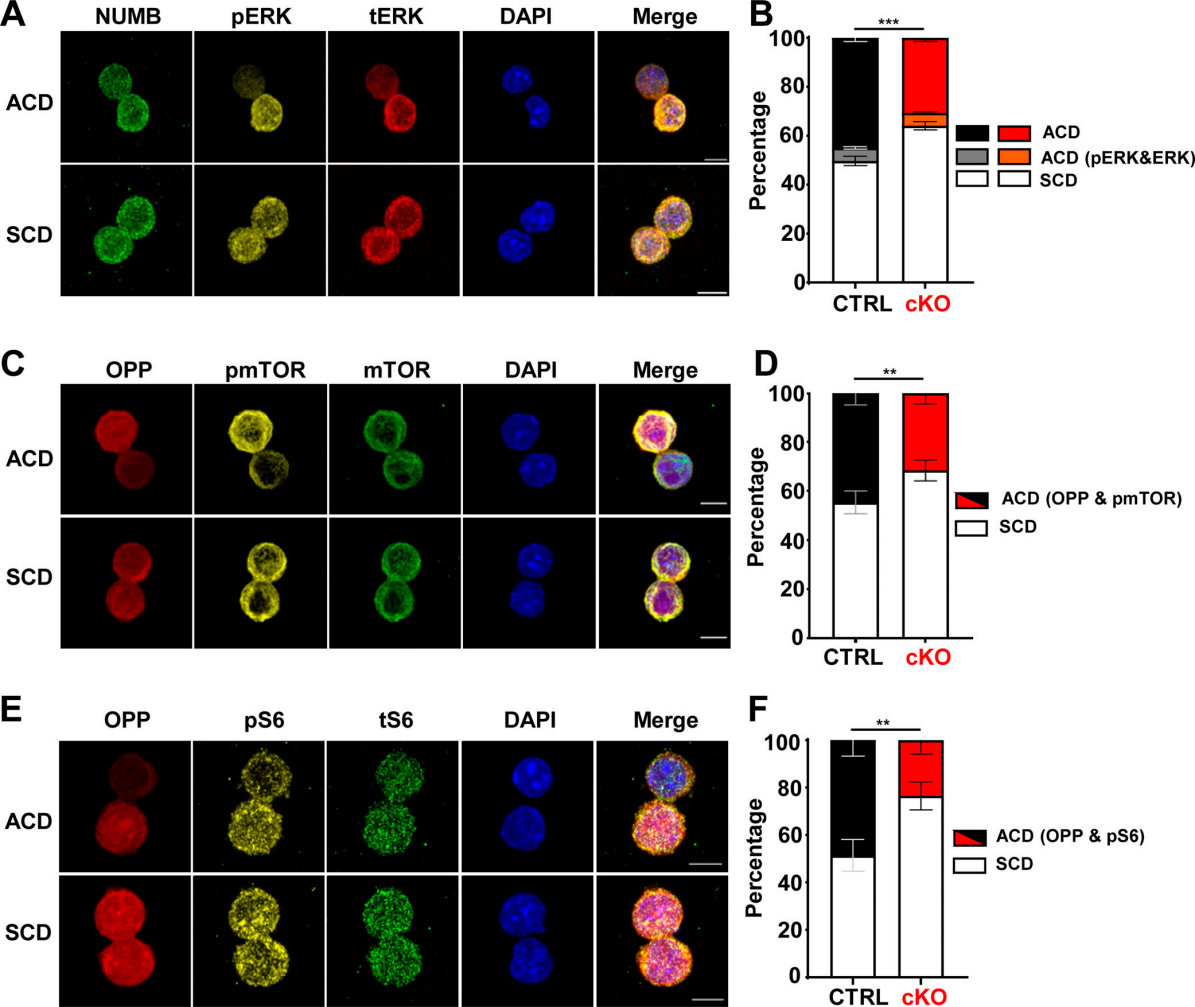
**Asymmetric distribution of active signaling molecules. (A, C, and E)** Representative immunofluorescence images showing the distribution of NUMB, pERK, and total ERK (A); OPP, pmTOR, and total mTOR (C); or OPP, pS6, and total S6 (E) in paired daughter HSCs (bar = 5 µm). **(B, D, and F)** Stacked bar charts depicting the percentage of asymmetric and symmetric divisions in paired daughter cells from CTRL (black) versus MEK1 cKO (red) HSC cultures for A, C, and E, respectively. Segregation of total mTOR and total S6 was not observed (100% SCD). The charts combine the results of three independent experiments (mean percentage ± SD; ≈200–250 cells total). Images were acquired on a Zeiss LSM 980 inverse confocal microscope using a Plan-Apochromat 63× oil objective (NA 1.4). **P < 0.01 and ***P < 0.001.

The correlation of NUMB expression with signaling activity observed in the FACS analysis suggested the intriguing possibility of an asymmetric segregation of signaling molecules during HSC division. Indeed, the levels of both total and pERK in paired daughter immunofluorescence assay were higher in NUMB^high^ cells (Fig. 4 A). Moreover, asymmetric segregation of ERK was reduced in MEK1-cKO HSCs (Fig. 4 B).

In contrast, pmTOR (S2448), but not mTOR, was asymmetrically segregated (Fig. 4 C), as suggested by the population analysis in Fig. S3 C. Similarly, pS6, but not S6, was asymmetrically segregated (Fig. 4 E), again in line with the FACS experiments in Fig. 2 B and Fig. S3 E. Together, these data indicate that at variance with ERK, which is asymmetrically segregated, mTORC1 is differentially activated in asymmetrically divided daughter cells, suggesting segregation of the signaling machinery necessary for its activation/inactivation. Importantly, mTORC1 activation coincided with high levels of translation (OPP staining) and NUMB. In contrast to translation, RNA transcription did not segregate asymmetrically (Fig. S4, E and F). These observations strongly suggest that the asymmetric activation of these signaling molecules labels differentiation-primed daughter cells. Consistent with all previous observations, the frequency of asymmetric segregation of pmTOR (S2448) (Fig. 4 D), pS6, and OPP (Fig. 4 F) was decreased in MEK1-cKO HSCs.

### Polar distribution of signaling molecules in premitotic HSCs

**Figure S5.**
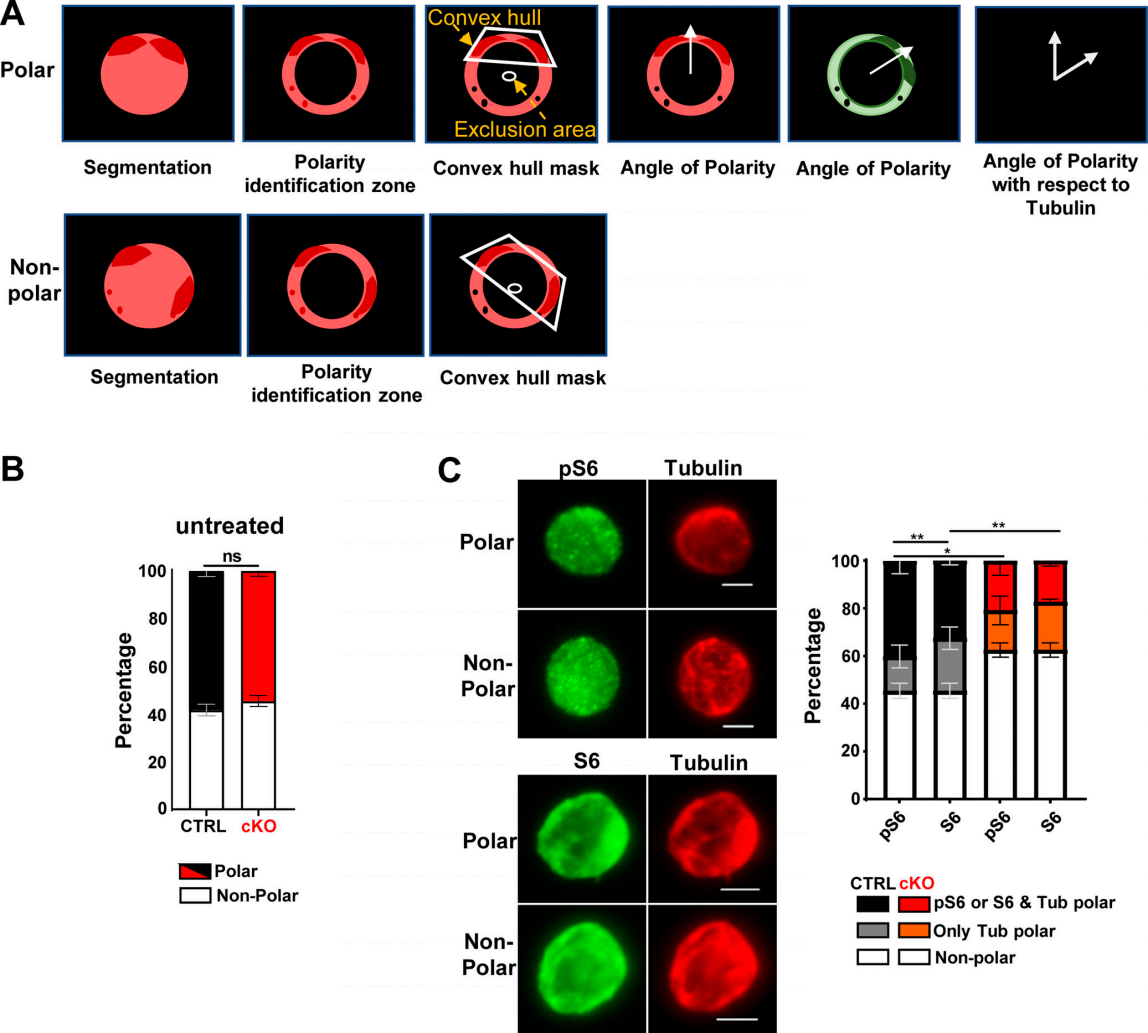
**Polar distribution of signaling molecules in HSCs.** Related to Fig. 5. **(A)** Cartoon representation of cell polarity analysis using a custom-made MATLAB code. Prior to MATLAB the files are subjected Fiji macros, which create z projections of the different channels and save single cells in Tif format. In MATLAB, a cell mask is generated with the maximum intensity projection from all the channels followed by background subtraction for individual channels. The “polarity identification zone” is set to 15 pixels from the cell boundary. K-means-based clustering of intensity into five bins is applied to identify the fluorescence intensity in the cell center and polarization zone. At this point, the number of clusters are quantified and speckles/bright spots smaller than 5 pixels are excluded. A circle with a radius of 10 pixels from the cell center is created, termed the “exclusion area.” A cell is defined polar if the convex hull of all the clusters identified does not overlap with the exclusion area. The average cluster intensity is quantified, and the cluster angle is defined with respect to the major axis of the cell. This process is repeated for all the available fluorescent channels. Finally, the polarization angle is defined as the angle between the direction of reference (tubulin) cluster and the direction of the cluster from different channel of the same cell. Unless otherwise specified, experiments were performed in triplicates, data were expressed as mean ± SD, and P values calculated using Student’s *t* test. Values below 0.05 were considered significant. Code available at: https://github.com/dhanlakshmi282/Matlab-code-polarity.git. **(B)** Percentage of cells with polar tubulin distribution in CTRL (black) and MEK1-cKO (red) HSCs isolated from untreated mice. **(C)** Representative immunofluorescence images of the distribution of tubulin and pS6 or S6 (bar = 3 μm). The stacked bar chart shows the results of three independent experiments (mean percentage ± SD; ≈300–350 cells total) conducted in CTRL (black) and in MEK1-cKO (red) recovering from 5-FU treatment. Images were acquired on a Zeiss Axio Observer 7 inverse microscope using a Plan-Apochromat 63× oil objective (NA 1.4). *P < 0.05 and **P < 0.01 according to unpaired *t* test comparing CTRL to cKO.

HSC polarity can be predictive of asymmetric division (Florian et al., 2018). We therefore tested whether the impact of MEK1 ablation on asymmetric HSC division correlated with defects in the distribution of tubulin (Florian et al., 2018) in HSCs in single-cell immunofluorescence followed by unbiased, automated localization analysis of tubulin distribution in FIJI and MATLAB (Fig. S5 A).

**Figure 5.**
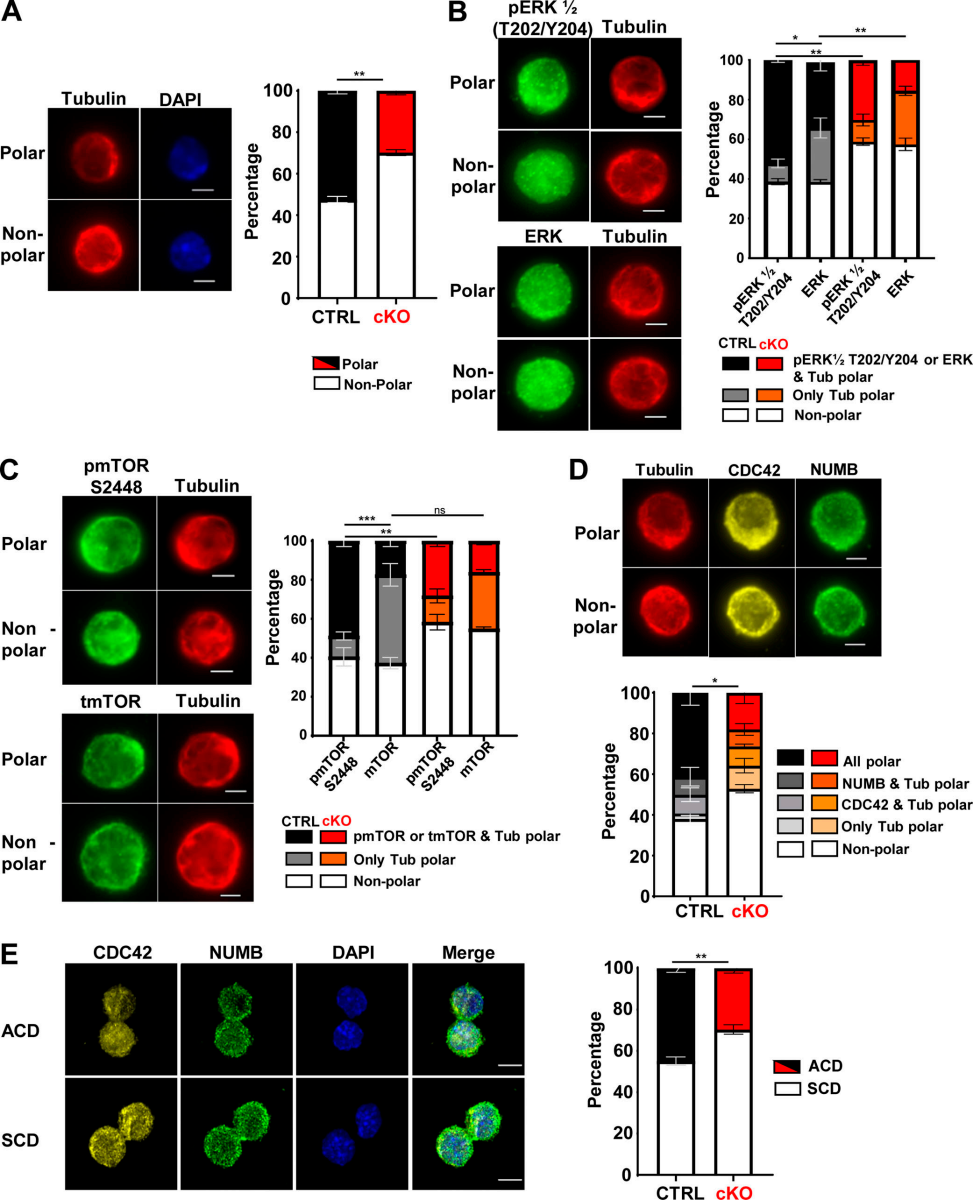
**Polar distribution of signaling molecules in premitotic HSCs. (A–D)** Representative immunofluorescence images of the distribution of (A) tubulin, (B) tubulin and pERK ½, or ERK, (C) tubulin and pmTOR S2448 or tmTOR, and (D) tubulin, CDC42, and NUMB in premitotic HSCs (bar = 3 µm). Images were acquired on a Zeiss Axio Observer 7 inverse microscope using a Plan-Apochromat 63× oil objective (NA 1.4). The stacked bar charts show the percentage of cells with polar tubulin and respective signaling molecules distribution in CTRL (black) and MEK1 cKO (red) HSCs. **(E)** Representative images showing the mitotic segregation of CDC42 and NUMB. The charts show percentage of cells with ACD from three independent experiments (bar = 5 µm; mean percentage ± SD; ≈300–400 cells total for polarity and ≈140–170 total cells for paired daughters) conducted in CTRL (black) and in MEK1 cKO (red) HSCs isolated from mice recovering from 5-FU treatment. Images were acquired on a Zeiss LSM 980 inverse confocal microscope using a Plan-Apochromat 63× oil objective (NA 1.4). *P < 0.05, **P < 0.01, and ***P < 0.001.

Consistent with the results of the paired daughter cell assay, MEK1 ablation strongly reduced the frequency of polar HSCs isolated from mice recovering from a single myeloablative injection (Fig. 5 A and Fig. S5 B). Remarkably, signaling molecules and in particular phosphorylated signaling molecules such as pERK (Fig. 5 B), pmTOR S2448 (Fig. 5 C), and pS6 (Fig. S5 C) also showed a polar distribution, although the frequency of cells with polarized tubulin was in general higher than that of HSCs with polarized activated signaling molecules. The frequency of HSCs with polarized signaling molecules was decreased in MEK1-cKO (Fig. 5, B and C; and Fig. S5 C).

These data suggested a connection between premitotic distribution and mitotic segregation of signaling molecules. To determine whether a similar link existed with cell fate determinants, we examined the relationship between the polarity-inducing small GTPase CDC42 (Florian et al., 2012) and NUMB, which has also been shown to be polarized in HSCs (Florian and Geiger, 2010) in premitotic and in mitotic cells. CDC42 and NUMB copolarized with each other and with tubulin to a large extent in both control and MEK1-cKO cells (Fig. 5 D). Notably, we also found that CDC42 cosegregated with NUMB during HSC division (Fig. 5 E). MEK1 ablation did not impact the colocalization of CDC42 and NUMB in premitotic or mitotic HSCs, but as observed before, it reduced the frequency of HSCs with copolarization/cosegregation of CDC42 and NUMB (Fig. 5, D and E).

### Signaling maintains HSC polarity and the balance between symmetric and asymmetric HSC division

**Figure 6.**
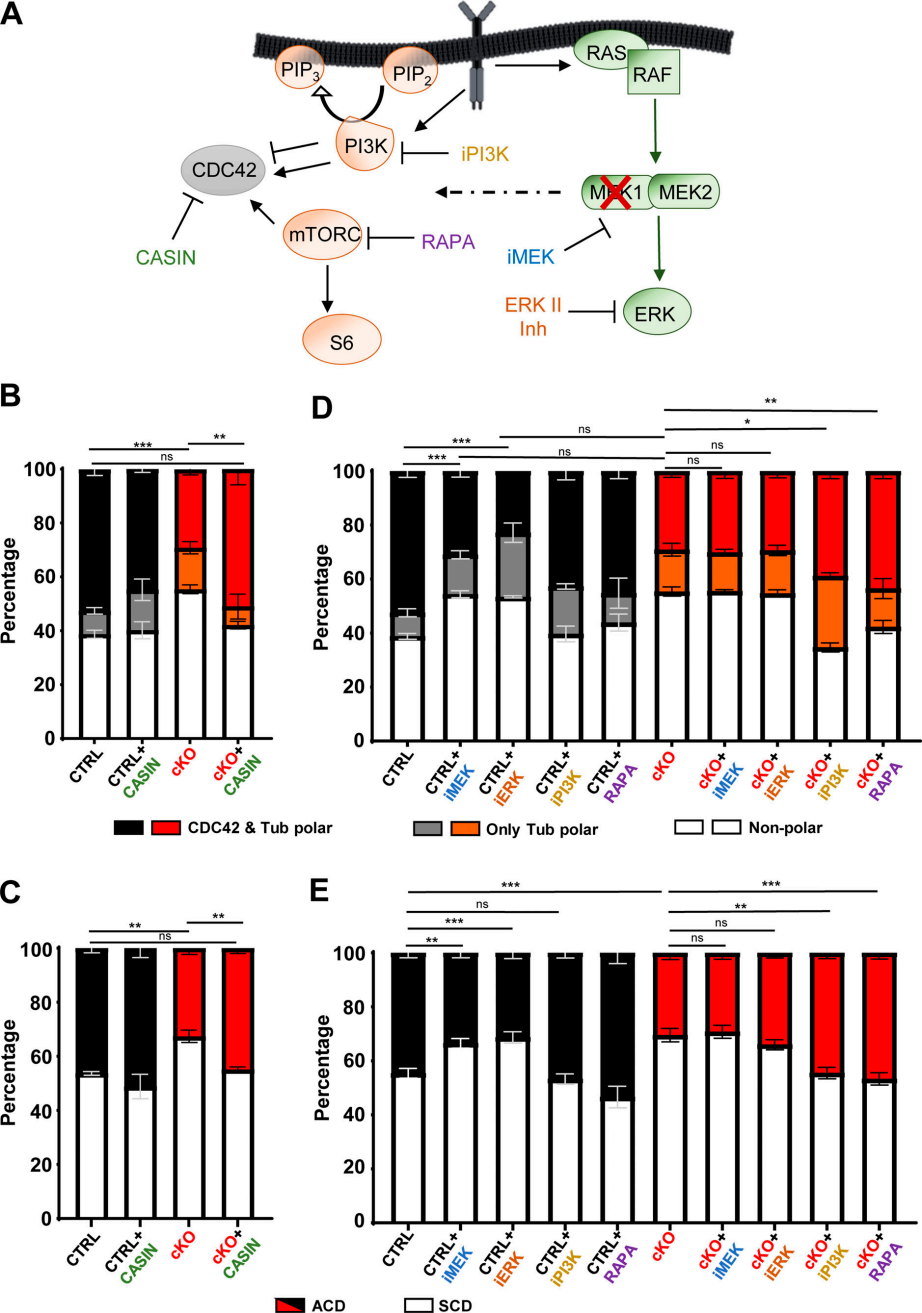
**MEK/ERK inhibition phenocopies, and PI3K/mTORC1 inhibition rescues, the cell division defects of MEK1 cKO HSCs. (A)** Pictogram showing the context of the signaling pathways and their inhibitors. MEK1 ablation or MEK/ERK inhibition increases the output of the PIP3/mTORC1 signaling pathway (broken arrow). **(B–E)** Stacked bar charts depicting the percentage of polarized HSC (B and D) or asymmetric divisions (C and E) in CTRL (black) versus MEK1 cKO (red) HSCs cultures isolated from 5-FU–injected mice and treated with the pharmacological inhibitors CASIN (10 µM), inhibitor of MEK (iMEK; U0126, 250 nM), inhibitor of ERK (iERK; ERK II inhibitor, 10 µM), iPI3K (LY294002, 1 µM), and Rapamycin (RAPA) (500 nM). The charts combine the results of three independent experiments (mean percentage ± SD). Polarity was assessed using CDC42 and tubulin as markers in ≈300–400 cells total; ACD/SCD was assessed using NUMB segregation as a marker in ≈200–250 cells total. *P < 0.05, **P < 0.01, and ***P < 0.001.

To gain further insight into the mechanism by which the signaling cascades regulated by MEK1 affect HSC polarity and mitotic behavior, we determined the impact of chemical inhibition on the phenotypes (Fig. 6 A). CDC42 activity influences HSC polarity, inversely correlates with the polarity of CDC42 itself (Florian et al., 2012), and negatively impacts the propensity of HSCs to undergo ACD (Florian et al., 2018). Inhibiting CDC42 with Casin rescued both the polarity of CDC42 and tubulin and the ACD (Fig. 6, B and C) phenotype of the MEK1-cKO cells, implying that MEK1 balances CDC42 activation in HSCs, and that maintaining this balance is necessary for both HSC polarity and ACD. In line with our previous results (Baumgartner et al., 2018), pharmacological inhibition of MEK or ERK in control HSCs phenocopied MEK1 ablation, decreasing the proportion of polarized as well as asymmetrically dividing HSCs (Fig. 6, D and E). Neither treatment had major effects on MEK1-cKO cells, confirming that the effect of the inhibitor is MEK1 dependent. Conversely, reducing PI3K or mTORC1 signaling in MEK1-cKO HSCs increased the frequency of polarized HSCs and asymmetric divisions, rescuing the phenotype. These treatments had no significant effect on the frequency of polar or asymmetrically dividing HSCs in control cultures (Fig. 6, D and E).

## Discussion

To date, the only protein implicated in the establishment of asymmetric HSC division was the dynein-binding protein Lis1, whose loss leads to asymmetric inheritance of the cell fate determinant NUMB and accelerated HSC differentiation (Zimdahl et al., 2014). Indeed, the very concept of ACD, well established in other cell types, has been controversial in the HSC field. While the dynamics of signaling events in HSCs are beginning to be unraveled (Wang et al., 2021), the relationship between signaling events and HSC cell fate decision, particularly at the level of ACD, has been difficult to tackle. Previous observations (Baumgartner et al., 2018; Rodgers et al., 2014) have indicated that a threshold of PI3K/mTORC1 signaling, determined by ERK-dependent feedback, increased the frequency of HSCs prone to differentiation. This led us to hypothesize that similar mechanisms might determine the fate of HSC daughter cells during the first division and that disabling the feedback will promote output (symmetric differentiation) over asymmetric division or symmetric self-renewal, giving us a handle to understand how asymmetry is established. To this aim, we deployed two complementary approaches. The FACS-based approach correlating the distribution of cell fate markers with the expression/activation levels of signaling molecules and/or organelles in HSC cultures allowed us to screen large numbers of cells and combinations. This is particularly important to offset the drawback posed by the heterogeneity of HSC populations, which, just as an example, might contain cells that differentiate without division (Grinenko et al., 2018). This overview provided a solid basis for in-depth investigation. The analysis of ACD at the single-cell level allowed us to directly visualize the behavior of the main molecular players involved. Importantly, both methods yielded fully consistent results, showing that ERK and mTORC1 signaling was higher in more differentiated cells (SCA1^low^, NUMB^high^), and that activated signaling molecules cosegregated with NUMB in the ACD assay. Interestingly, both methods also showed that ERK activation in daughter cells was increased mainly due to segregation of the protein; while mTORC1 activation, which also correlated with translation, compartmentalization was achieved by differential phosphorylation of mTOR and S6 in daughter cells that received the same amount of protein. Through perturbation of ERK, PI3K, and mTORC1 signaling we could show that these molecules are not only segregated/activated asymmetrically, but that their activity also plays a role in determining the frequency of symmetrically and asymmetrically dividing cells. Specifically, inhibiting MEK and ERK reduced the frequency of ACDs in control, but not MEK1-cKO cultures, while inhibiting PI3K or mTORC1 rescued it.

Collectively, the data show that signaling levels, regulated through spatial segregation or activation of signaling molecules, play a major role in determining HSC cell fate already at the stage of the first ACD. In particular, the MEK1/ERK module acts as a rheostat regulating both its own activation and the activation of PI3K and mTORC1. This is in line with our previous observations showing the importance of this feedback circuit in vivo (Baumgartner et al., 2018), as well as with reports that mTORC1 (Chen et al., 2008; Kharas et al., 2010b; Rodgers et al., 2014; Yilmaz et al., 2006) inhibition by rapamycin represents a good strategy to rejuvenate adult stem cell compartments (Neves et al., 2017).

We also found that the frequency of ACD correlates with, and is possibly predetermined by, premitotic polarity of signaling molecules. This has been proposed before based on the correlation of the polar distribution of CDC42 or the epigenetic marker histone 4 acetylated on lysine 16 in premitotic with ACD in young HSC (Florian et al., 2012, 2018). We have now determined that activated ERK and mTOR are distributed in a polar manner, although they do not completely copolarize with tubulin, and that the same rheostat centered on the MEK1/ERK module and its effects on PI3K signaling also control premitotic polarity as determined by the distribution of tubulin and CDC42.

Disabling the feedback by genetic ablation of MEK1 or by chemical inhibition of MEK/ERK in control HSCs reduces the frequency of cells showing polar distribution of both molecules, while treatment with CASIN rescues the polarity and ACD defects of MEK1-cKO cells. The link between these two processes is further strengthened by the copolarization and cosegregation of CDC42 and NUMB in premitotic and mitotic cells.

To date, CDC42 activity and CDC42-induced polarity of HSC are known to be regulated by external signals such as WNT or osteopontin (Florian et al., 2013; Guidi et al., 2017) or by YAP/TAZ/Scribble (Althoff et al., 2020). We now show that PI3K and mTORC1 inhibition cause the repolarization of CDC42, and by inference reduce its activation. The relationship between PI3K and CDC42 is complicated and regulated by both positive and negative feedback loops. PI3K has been shown to activate CDC42, Rac, and RhoG. These GTPases, in turn, activate PI3K, but only in combination, establishing a cooperative positive feedback loop (Yang et al., 2012). Reduced CDC42 activation could be the basis of the increase in polarity observed in iPI3K-treated cKO cells. On the other hand, PIP3 generated by PI3K could also limit CDC42 activation by activating GAPs (GTPase activating proteins) for Rac and CDC42 (Schlam et al., 2015). PIP3 binding by CDC42-GAP (Krugmann et al., 2002) could also restrict inactivation to specific plasma membrane domains, which would fit very well with the role of CDC42 inactivation in establishing polarity. In this case, inhibiting PI3K would activate CDC42, decreasing its polar distribution as observed in the control cells in Fig. 6 B. In any case, this activation would be below the threshold necessary to decrease tubulin polarity because this aspect is not affected by the inhibitor.

mTORC1 inhibition also rescues CDC42 and tubulin polarity in cKO cells. The underlying mechanism is unclear, but the mTORC1 inhibitor used in this study, rapamycin, also rescues the HSC exhaustion phenotype of MEK1-cKO MEK1 mice (Baumgartner et al., 2018), the polarity defects in TSC2 knocked down neurons in culture (Choi et al., 2008), and, if administered to presymptomatic mice, can suppress the loss of neuronal polarity caused by phosphatase and tensin homolog ablation in vivo (Zhou et al., 2009).

Although it remains unclear how exactly polarity and ACD are established, our data imply that mTORC1 and translation regulate this process. In this context, it is important to note a possible role for RNA-binding proteins such SYNCRIP, which is essential to maintain HSC polarity by promoting the translation of CDC42 (Herrejon Chavez et al., 2023) or MUSASHI2, previously shown to inhibit NUMB translation (Hope et al., 2010; Ito et al., 2010; Kharas et al., 2010a) to promote stemness. Finally, although we could not find evidence for asymmetric transcriptional activity in dividing cells (Fig. S4 E), we have shown that SCA1^low^ cells generated in culture expressed higher amounts of all proteins studied (Fig. S3 B). It is possible that this is due to differential segregation or activation of transcriptional and epigenetic regulators controlling their expression, as it has been shown for the control of NUMB and MYC by SATB1 (Will et al., 2013).

Our data indicate that signaling molecules, and more specifically their premitotic polarization and their spatial segregation, drive ACD in HSC and that fine-tuning signaling is key to maintaining HSC polarity and ACD. The results advance our understanding of how asymmetry is established in HSC division. It is important to point out that although the picture we paint is based on snapshots, it shows the statistically most likely situation, but does not include signaling dynamics, which are likely important in determining both polarity and ACD. The study of signaling dynamics in HSCs is complicated by the extremely poor transfection efficiency of HSCs in general, and of mouse HSCs in particular, hindering the use of signaling reporters (Wang et al., 2021). Furthermore, the existing signaling reporters would not be helpful in determining the position of the activated signaling molecules in premitotic cells.

On a translational note, the transition from HSC self-renewal to differentiation in multipotent progenitors with intermediate or short-term reconstitution potential is the rate-limiting step after which the HSC progeny is rapidly amplified to meet the demands of hematopoiesis. This crucial step is impaired during aging, with the HSC pool expanding through symmetric self-renewing divisions without any contribution to differentiation (Bernitz et al., 2016; Florian et al., 2018). Single-cell RNA sequencing (Kowalczyk et al., 2015) as well as comprehensive epigenomic profiling (Sun et al., 2014) are also consistent with an increased propensity of aged HSCs toward self-renewing rather than asymmetric divisions giving rise to differentiated blood cells. MEK1 ablation and MEK/ERK inhibitors, in clinical development/use as anticancer drugs (Moore et al., 2020), modulate HSC differentiation at the level of asymmetric division and could be developed into a strategy to restore output from dysfunctional, “old” HSCs.

## Materials and methods

### Mice

MEK1F/F;Vav-Cre2^+^ mice (Baumgartner et al., 2018) were maintained on a C57/B8 background and housed under specific pathogen–free conditions at the Max Perutz Laboratories in Vienna (Permit: GZ66.006/0005-V/3b/2018). Where indicated, 8–16-wk-old mice were treated with 5-FU (Sigma-Aldrich) to induce emergency hematopoiesis and sacrificed 9 days after (Baumgartner et al., 2018).

### HSC isolation and culture

For FAC sorting, bone marrow (BM) cells were first stained with the biotinylated lineage cocktail (Table 1) followed by lineage depletion using α-biotin beads and an AutoMACS cell separator (Miltenyi Biotec). Lineage-depleted BM cells were then labeled with a panel of antibodies to identify HSCs (Table 1). Mouse HSCs were defined as follows: lin^−^ Sca1^+^ c-KIT^+^ CD150^+^ CD48^−^. HSCs were cultured StemSpan (StemCell Technologies) medium supplemented with SCF (10 ng/ml; PeproTech) and TPO (10 ng/ml; PeproTech).

**Table 1.** Antibodies used in the study


**Antibodies used for lineage depletion**					
Antigen	Dilution	Tag	Company	Clone	
Anti-Mouse CD5	1:100	Biotin	eBioscience	53-7.3	
Anti-Mouse CD3e	1:100	Biotin	eBioscience	145-2C11	
Anti-Mouse TER-119	1:100	Biotin	eBioscience	TER-119	
Anti-Mouse CD8a	1:100	Biotin	eBioscience	53-6.7	
Anti-Mo/Hu CD45R (B220)	1:100	Biotin	eBioscience	RA3-6B2	
Anti-Mouse Ly-6G (Gr-1)	1:100	Biotin	eBioscience	RB6-8C5	
Anti-Mouse CD2	1:100	Biotin	eBioscience	RM2-5	
Anti-Mo/Hu CD11b	1:100	Biotin	BioLegend	M1/70	
**Antibodies used for HSC sorting**					
Antigen	Dilution	Fluorochrome	Company	Clone	
Streptavidin	1:100	PerCP	BD Pharmigen	554064	
Anti-Mouse Ly-6A/E (Sca-1)	1:100	BV 510	BioLegend	D7	
Anti-Mouse CD150 (signaling lymphocytic activation molecule)	1:100	BV 5605	BioLegend	TC15-12F12.2	
Anti-Mouse CD117 (C-Kit)	1:100	APC-eFluor 780	Invitrogen	2B8	
Anti-Mouse CD48	1:100	PE/Cy7	BioLegend	HM48-1	
**Primary antibodies used for cultured HSC analysis**					
Antigen	Dilution	Fluorochrome	Company	Clone	
Anti-Mouse Ly-6A/E (Sca-1)	1:100	FITC	BioLegend	D7	
Anti-Mouse EPCR	1:100	PE	BioLegend	RCR-40	
**Primary antibodies used for immunostaining**					
Host	Antibody	Dilution	Company	Cat. No.	
Goat	NUMB	1:1,600	Abcam	Ab4147	
Mouse	pERK (T202/Y204)	1:400	Cell Signalling	9106S	
Rabbit	ERK	1:400	Cell Signalling	9102	
Rabbit	pS6	1:1,200	Cell Signalling	5364S	
Mouse	S6	1.400	Cell Signalling	2317S	
Rabbit	mTOR S2448	1:200	Cell Signalling	5536S	
Mouse	mTOR	1:200	Proteintech	66888-1	
Rat	Tubulin	1:1,200	ORIGENE	SM568P	
Rabbit	CDC42	1:100	Merck Millipore	07–1,466	
**Secondary antibodies used for immunostaining**					
Host	Antigen	Dilution	Company	Cat. No.	Fluorochrome
Donkey	Anti-Goat	1:800	Invitrogen	A11055	Alexa Fluor 488
Donkey	Anti-Rat	1:800	Invitrogen	A21208	Alexa Fluor 488
Donkey	Anti-Rabbit	1:800	Invitrogen	A10040	Alexa Fluor 546
Donkey	Anti-Mouse	1:800	Invitrogen	A31571	Alexa Fluor 647

### In vitro long-term co-culture assay

For mouse HSCs (LSK CD150^+^ CD48^−^), stromal feeder layer cells (OP9) were plated in a flat-bottom, tissue culture–treated plate according to the protocol. After mitomycin-C treatment of the feeder layers, 100 HSCs per well plated onto the feeder layer and incubated for up to 10 wk at 33°C (5% CO_2_) in MyeloCult medium (M5300 from, supplemented with 1 μM hydrocortisone; StemCell Technologies). Half of the medium was replaced with fresh medium once per week. Cells were harvested and transferred to a cytokine-supplemented methylcellulose medium (MethoCult GF M3434; StemCell Technologies). Colony forming units (CFUs) were scored after 10 days.

### FACS analysis

HSCs were incubated for 20 min at 37°C in CellTrace Violet (Invitrogen), resuspended in 1 ml PBS containing 10% BSA for 10 min, washed, and placed in culture. For intracellular staining for (phospho)-proteins, cells were fixed for 20 min at RT in Cytofix/Cytoperm buffer (BD Bioscience), washed twice with PermWash buffer (BD Bioscience), and incubated for 1 h at RT with the primary antibodies (Table 1). Cells were washed thrice in PermWash buffer and subsequently incubated with the conjugated secondary antibodies (Table 1) for 1 h at RT. After washing three times in PermWash buffer, cells were resuspended in 2% BSA/PBS and analyzed. Cells were analyzed on a BD LSM Fortessa analyzer. For metabolic and organelle analyses, cells were incubated for 20–30 min at 37°C with respective dyes (Table 2), according to manufacturer’s instructions, together with verapamil (50 µM, #V4629; Sigma-Aldrich). Unbiased clustering of data was performed using the FlowSOM plugin from FlowJo (Van Gassen et al., 2015). The number of clusters was arbitrarily set to a total of three. Statistical analysis was performed using Fisher’s exact test.

**Table 2.** Fluorescent dyes used in the study

Dyes	Dilution	Source
MitoTracker Green	30 nM	Thermo Fisher Scientific
TMRE	100 nM	Thermo Fisher Scientific
LysoTracker	50 µM	Thermo Fisher Scientific
CellROX	2.5 µM	Thermo Fisher Scientific

### Immunofluorescence

To determine the numbers of ACDs, cells plated on fibronectin-coated sterile cover glasses were treated with Nocodazole (10 nM; Sigma-Aldrich) after 16 h of culturing to synchronize mitotic events and were cultured for 40 h at 37°C in 5% CO_2_ (Cheng et al., 2019). Polarity experiments were performed following a previously established protocol (Florian et al., 2012). Where indicated, cells were cultured in the presence of chemical inhibitors (Table 3). FAC-sorted HSCs were plated onto fibronectin-coated coverslips. After culturing, cells were fixed in PBS/4% PFA or BD cytofix for 20 min at RT. Cells were permeabilized in PBS/0.1% Triton X-100 for 10 min at RT prior to blocking in PBS/0.1% Triton/5% BSA for 30 min at RT. Cells were incubated in blocking solution with the primary Abs for 1 h at RT. After incubation, cells were washed three to five times with PBS and incubated for 1 h at RT in blocking solution with secondary antibodies (Table 1). Cells were then washed thrice in PBS and mounted in VectaShield with DAPI (Vector Laboratories) and further inspected using an epifluorescence (Zeiss Axio Imager Z2) or a confocal microscope (LSM980-AiryScan). Global transcription (Click-iT RNA Alexa Fluor 488 Imaging Kit, Cat#C10329; Thermo Fisher Scientific) or translation (Click-iT Plus OPP Alexa Fluor 647 Protein Synthesis Assay Kit, Cat#C10458) rate was analyzed in vitro using Click-iT chemistry according to manufacturer’s instructions.

**Table 3.** Chemical inhibitors used in the study

Inhibitors	Dilution	Source
Casin	10 nM	Geiger lab
MEK U0126	250 nM	Cell Signalling
ERK II inhibitor	10 µM	Calbiochem
Pi3K LY294002	1 µM	Cell Signalling
Rapamycin	500 nM	Enzo Life Science

### Quantitative RT-PCR

RNA was extracted from sorted cultured cells using the RNA isolation kit (Macherey-Nagel), and cDNAs were synthesized using Lunascript RT supermix (NEB). Quantitative PCR (qPCR) was performed using Luna Universal qPCR Master Mix (NEB) according to the manufacturer’s instructions using a Mastercycler realplex real-time PCR system (Eppendorf). All primer sequences are listed in Table 4. mRNA levels were calculated and normalized to *Gapdh* housekeeping gene using the Δ*C*T method.

**Table 4.** qPCR primers used in the study


*Prdm16*	Forward sequence	5′-ATC​CAC​AGC​ACG​GTG​AAG​CCA​T-3′
	Reverse sequence	5′-ACA​TCT​GCC​CAC​AGT​CCT​TGC​A-3′
*Fstl1*	Forward sequence	5′-CTG​TCT​GAT​GAG​AAC​GCT​GAC​TG-3′
	Reverse sequence	5′-AGA​CAC​AGC​GAT​TGC​AGT​CCA​C-3′
*Prex2*	Forward sequence	5′-ACA​CAT​GCC​AGT​GTC​ATC​GCA​C-3′
	Reverse sequence	5′-CCT​GAG​CAC​TTT​CAA​GAC​TGT​CG-3′
*Mpdz*	Forward sequence	5′-CAC​TCG​CAG​TTA​CCA​AGT​AGC​C-3′
	Reverse sequence	5′-GAC​AGA​CTC​AGC​ATC​AGA​TGC​C-3′
*Cebpa*	Forward sequence	5′-GCA​AAG​CCA​AGA​AGT​CGG​TGG​A-3′
	Reverse sequence	5′-CCT​TCT​GTT​GCG​TCT​CCA​CGT​T-3′
*Siglecf*	Forward sequence	5′-CTC​CAC​AGA​AGA​TGA​CCA​TCA​GG-3′
	Reverse sequence	5′-CTG​TCA​GCC​ATA​CAG​ACC​AGG​C-3′
*Klhl4*	Forward sequence	5′-CTT​GCC​TGA​GAG​AAG​GTC​CAT​G-3′
	Reverse sequence	5′-CCA​ACT​GTT​GGT​TCG​GAG​GTC​A-3′
*Ptk2*	Forward sequence	5′-ACA​TCA​AGG​CGT​GTA​CCT​GAG​C-3′
	Reverse sequence	5′-GTG​AGG​ATG​GTC​AAA​CTG​ACG​C-3′
*Gapdh*	Forward sequence	5′-CAT​CAC​TGC​CAC​CCA​GAA​GAC​TG-3′
	Reverse sequence	5′-ATG​CCA​GTG​AGC​TTC​CCG​TTC​AG-3′

### Antibodies, fluorescent dyes, and chemical inhibitors

A list of the antibodies used in this study is provided in Table 1. Fluorescent dyes are listed in Table 2, and chemical inhibitors in Table 3.

### Data analysis and statistical methods

#### Paired daughter assay

Images were analyzed and processed using the Fiji software (Image J). Sister-cell ratios were calculated by dividing the sum of pixel fluorescence intensities of one daughter cell by the other. Sister cell ratios of >1.5× and <1.2× were considered indicative of asymmetric or symmetric division, respectively.

#### Polarity

We have developed a pipeline consisting of Fiji macros and a MATLAB script that extracts polarity information from the single cells. A detailed description of the pipeline is in the legend to Fig. S5. Images were acquired on a Zeiss LSM 980 inverse confocal microscope and Zeiss Axio Observer 7 inverse microscope using a Plan-Apochromat 63× oil objective (NA 1.4). Statistical analysis was performed using the two-tailed Student’s *t* test.

### Online supplemental material

Fig. S1 shows the characteristics of HSCs analyzed in this study. Fig. S2 shows the MEK1 ablation increases the frequency of metabolically active HSCs generated in culture. Fig. S3 shows that the MEK1 ablation increases the frequency of HSCs with higher signaling activity. Fig. S4 shows the frequency of paired daughter cells and transcriptional activity in HSC doublets. Fig. S5 shows the polar distribution of signaling molecules in HSCs.

## Data Availability

The data is available from the corresponding author upon reasonable request.
